# Tobacco and nicotine products in Switzerland: a national analysis of market notifications

**DOI:** 10.3389/fpubh.2026.1876332

**Published:** 2026-10-28

**Authors:** Jérémy Cros, Karin Zürcher, Luc Lebon

**Affiliations:** Department of Health Promotion and Prevention, Unisanté, University Center for Primary Care and Public Health & University of Lausanne, Lausanne, Switzerland

**Keywords:** cigarette, cigars, electronic nicotine delivery systems, nicotine pouches, public health surveillance, tobacco control, tobacco industry, tobacco products

## Abstract

Since the mid-2000s, the tobacco and nicotine products market has undergone rapid diversification. Since 2024, the Federal Act on Tobacco Products modernized the Swiss product notification system through the mandatory use of the electronic platform Tabacinfo.ch. This study aims to describe the scope, evolution and characteristics of tobacco and nicotine products notified on the Swiss market as of 2026. The number of notified products increased from 2,460 in 2000 to 9,857 in January 2026. Electronic cigarettes represent 51% of all notifications (*n* = 5,032), followed by smoking tobacco products (40%; *n* = 3,956), other products (6%; *n* = 590), nicotine products for oral use (2%; *n* = 200) and heated tobacco products (1%; *n* = 79). Cigars are the most frequently notified product (*n* = 3,102). Cigarette notifications remained limited, reflecting a market concentrated around major tobacco companies and supermarket brands. Flavor descriptors among electronic cigarettes, nicotine pouches and snus are dominated by cooling-, mint- and fruit-related terms. Several descriptors that may be particularly appealing to young people were identified (e.g. lemonade, cola and candy). Most nicotine-containing electronic cigarettes are concentrated between 17 and 20 mg/mL, whereas nicotine pouch concentrations vary widely from 1.5 to 30 mg per pouch. Switzerland remains a distinctive case in Europe, with comparatively weak regulations that may facilitate the availability and attractiveness of a wide range of addictive nicotine products. These results document products on the market and may inform policymaking. Strengthening tobacco control measures, particularly regarding flavors, nicotine concentration and product-specific exemptions, would reduce exposure to harmful products and limit their appeal, especially among young people.

## 1 Introduction

Tobacco use remains the leading risk factor for non-communicable diseases and the main preventable cause of death (1). Since the mid-2000s, the nicotine market has undergone rapid diversification, with the emergence of numerous novel products alongside conventional combustible tobacco (2–5). Driven by the expansion of the range of flavors (6), the diversity of nicotine delivery methods and intensive commercial marketing, this transformation has raised new concerns about initiation and addiction, particularly among young people (7, 8).

Switzerland occupies a distinctive position within this evolving tobacco control landscape. Long perceived as one of the most permissive regulatory environments in Europe, the country has been characterized by comparatively weak tobacco control measures and described as a favorable setting for tobacco industry activities (2). This permissiveness is closely linked to Switzerland’s longstanding failure to ratify the World Health Organization Framework Convention on Tobacco Control, as well as to the documented influence of the tobacco industry on public policymaking processes (9). Within this regulatory context, the Swiss market represents a strategic context for the introduction of novel nicotine products and innovative commercial strategies. This illustrates how regulatory permissiveness may support product development and diversification.

A major regulatory shift occurred in October 2024 with the entry into force of the Federal Act on Tobacco Products (2, 10, 11). Although manufacturers and importers were already required to declare the tobacco and nicotine products placed on the Swiss market under the previous regulations, the new law modernized this process by introducing mandatory use of a national electronic platform Tabacinfo.ch (article 26). For the first time, this public database provides a standardized source of information on tobacco and nicotine products marketed in Switzerland, enabling systematic market surveillance. A similar notification system exists in the European Union through the Common Entry Gate (EU-CEG) established under the Tobacco Products Directive (12). The present study therefore aims to describe and analyze the scope, evolution and characteristics of tobacco and nicotine products notified on the Swiss market. By examining the notified product landscape, this study seeks to document market dynamics, support regulatory evaluation and inform evidence-based tobacco control policies.

## 2 Method

The data were extracted by the Federal Office of Public Health from the Tabacinfo.ch platform on 15 January 2026. For each product, manufacturers and importers are required to specify its name, type (see the categories below), the nicotine concentration (mg/mL) for products related to the *electronic cigarette* category, the date of launch on the market (which can be retrospective) and the date of modification, if applicable. A reported market launch date was available for 76% of notified products (*n* = 7,447). Products without a reported market launch date (*n* = 2,410; 24%) were retained in all descriptive analyses of products notified as of January 2026 but were not included in analyses of temporal trends. Historical analyses were based on the market launch date reported by manufacturers and importers.

The data were used as provided by the manufacturers and importers on the platform. They may contain errors or missing data. They were not individually compared to the products actually available on the Swiss market. Each record contained in the database was considered as a distinct product (even when they differ only by flavor or nicotine level). No additional deduplication procedure was applied. Products were analyzed as provided in the database.

To facilitate the interpretation of this analysis, the products were grouped as follows:

- *Tobacco products*: cigarettes, cigars, cigarillos, herbal cigarettes, pipe tobacco, roll-your-own tobacco and water pipe tobacco;
- *Heated products*: heated tobacco products (listed as “tobacco stick” in the database) and herbal products for heating without tobacco;
- *Electronic cigarettes*: disposable cartridge/pod with/without nicotine, electronic cigarette with/without nicotine and refill liquid with/without nicotine;
- *Nicotine products for oral use*: chewing tobacco, nicotine pouches (listed as “nicotine products for oral use without tobacco” in the database) and snus;
- *Other products*: cannabis with low tetrahydrocannabinol (THC) content and snuff with tobacco.

These analytical categories were created by the authors to facilitate interpretation and do not exactly correspond to legal categories defined in the Federal Act on Tobacco Products.

Descriptive statistics (number, percentage and mean) were calculated using Microsoft Excel (Microsoft Corp., Redmond, WA, USA). To characterize flavor descriptors, product names from electronic cigarettes, nicotine pouches and snus were analyzed. They were automatically separated into individual words and number of occurrences were calculated. Words were subsequently reviewed manually by one investigator to identify recurring descriptors. Product names were analyzed in their original language. For electronic cigarette products, flavor descriptors were additionally compared according to nicotine content (with versus without nicotine). Given the descriptive nature of the analysis and the fact that the manual review was conducted by a single investigator, no formal inter-rater reliability assessment was performed.

## 3 Results

### 3.1 Overview

The number of products notified on the Swiss market rose steadily, reaching its highest level in 2026. Among products with a reported market launch date, an average of 140 products were introduced each year between 2014 and 2021 (Figure 1A). The number of products with a reported launch date then increased substantially: +375 in 2022, +1,118 in 2023, +1,971 in 2024 and +2,428 in 2025. The highest number of products with a reported market launch date occurred in:

- 2022 for electronic cigarettes without nicotine (+22);
- 2023 for cigars (635 products) and cannabis with low THC content (+104);
- 2024 for cigarettes (+33), roll-your-own tobacco (+18), electronic cigarettes with nicotine (+185) and refill liquid with nicotine (+876);
- 2025 for cigarillos (+42), pipe tobacco (+82), nicotine pouches (+65), snus (+15), refill liquid without nicotine (+806), disposable cartridges/pods with nicotine (+380) and snuff with tobacco (+9).

**Figure 1:**
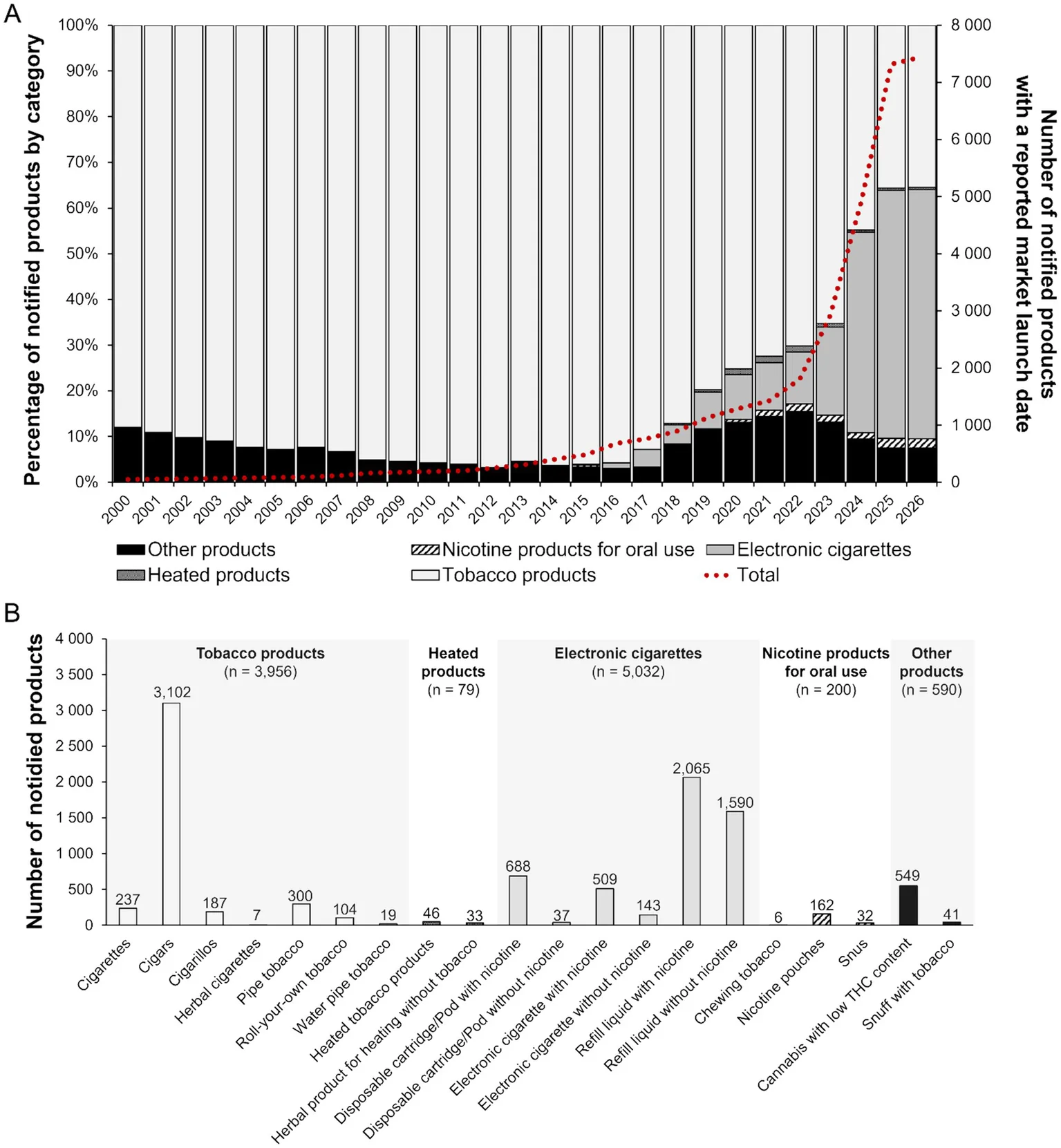
(A) Distribution of notified tobacco and nicotine products by analytical category according to their reported market launch year (2000–2026), together with the total number of notified products with a reported market launch date per year (dotted red line). A reported market launch date was available for 7,447 products (76%), whereas 2,410 products (24%) had no reported market launch date and were therefore not included in the temporal trend analysis. (B) Notified tobacco and nicotine products recorded in the Tabacinfo.ch database as of January 2026 (*n* = 9,857). Data were extracted from the official Swiss database Tabacinfo.ch. Products were grouped into analytical categories for readability: Tobacco products include cigarettes, cigars, cigarillos, herbal cigarettes, pipe tobacco, roll-your-own tobacco and water pipe tobacco; heated products include heated tobacco products and herbal products for heating without tobacco; electronic cigarettes include disposable cartridge/pod with/without nicotine, electronic cigarette with/without nicotine and refill liquid with/without nicotine; nicotine products for oral use include chewing tobacco, nicotine pouches and snus; other products include cannabis with low THC content and snuff with tobacco.

These recent arrivals have reshaped the Swiss market for tobacco and nicotine products (Figure 1A).

In January 2026, 9,857 different products were marketed by 121 companies (Figure 1B). Of these, 5,032 are electronic cigarettes (51%), 3,956 are smoking tobacco products (40%), 590 are other products (6%), 200 are nicotine products for oral use (2%), 79 are heated products (1%). The most prevalent products were cigars (3,102; 31%) and refill products with (2,065; 21%) and without nicotine (1,590; 16%).

The five companies that notified the most products are Playsure SA (*n* = 1,762, including 889 refill liquids with nicotine and 668 refill liquids without nicotine), InSmoke AG (*n* = 744, including 408 refill liquids with nicotine), The Royal Cigar Company AG (*n* = 627, including 547 cigars), Oettinger Davidoff AG (*n* = 541, including 366 cigars) and Intertabak AG (*n* = 533, including 466 cigars). The Supplementary Table 1 presents in detail the three companies that recorded the highest number of products by category as of January 2026.

### 3.2 Products notified by BAT, PM, and JTI

Among the 3 major tobacco companies, British American Tobacco (BAT) recorded the highest number of products (*n* = 185), followed by Philip Morris (PM; *n* = 105) and Japan Tobacco International (JTI; *n* = 44). Cigarettes remain the largest product category for all 3 companies (Figure 2). While BAT and JTI portfolios were largely dominated by cigarettes, PM showed a more diversified profile with a greater proportion of heated tobacco products. Beyond cigarettes, JTI’s market is essentially focused on roll-your-own tobacco products. No heated tobacco products attributed to JTI were identified in the database despite the company’s presence in this market segment. Several supermarkets are also positioned in the smoking tobacco market, such as Denner (*n* = 15 for cigarettes and *n* = 1 for roll-your-own tobacco), Coop (*n* = 11 for cigarettes and *n* = 1 for roll-your-own tobacco) and Lidl (*n* = 4 for cigarettes).

**Figure 2:**
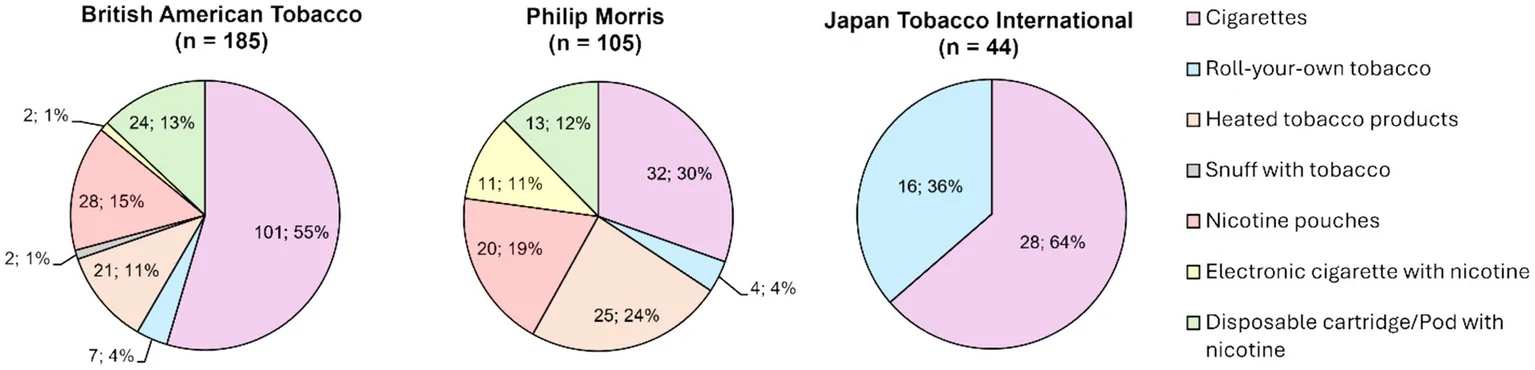
Distribution of notified tobacco and nicotine products among British American Tobacco, Philip Morris and Japan Tobacco International (data from the official database Tabacinfo.ch, as of January 2026). Percentages indicate the proportion of notified products within each company’s portfolio.

### 3.3 Flavors and nicotine concentrations for electronic cigarettes, nicotine pouches and snus

Regarding electronic cigarettes, nicotine pouches and snus, a descriptive analysis of flavor descriptors was conducted to determine the frequency of appearance of adjectives used to describe the products in their notified names. The most used adjectives refer to the cooling effect of the products (1,328 occurrences among 5,227 products; 25%). The adjective *ice* occurs most frequently, with 746 occurrences (14%). In terms of flavors, the most marketed flavor is mint (437 occurrences; 8%), followed by strawberry (404 occurrences; 8%), lemon (288 occurrences; 6%), raspberry (264 occurrences; 5%), watermelon (248 occurrences; 5%) and kiwi (210 occurrences; 4%). Tobacco flavor is mentioned in 181 occurrences (3%). Several products also feature flavors that may be particularly appealing to young people, such as lemonade (102 occurrences; 2%), cola (98 occurrences; 2%), candy (32 occurrences; 1%), bubble gum (29 occurrences; 1%), mojito (22 occurrences; <1%) and cookies (9 occurrences; <1%). Among electronic cigarette products, flavor descriptors seem generally more frequent in products with nicotine (*n* = 3,262) than in those without nicotine (*n* = 1,770). Overall, three out of four products have English-language names and one out of five have French names. The use of German and Italian words for product names remains anecdotal.

Among notified electronic cigarettes with nicotine, 96% of products have a nicotine concentration between 17 and 20 mg/mL. For refill liquids, the average nicotine concentration is 14 mg/mL (39% of products between 10 and 19 mg/mL and 42% at 20 mg/mL). Finally, disposable cartridges/pods have an average nicotine concentration of 17 mg/mL (23% of products between 10 and 19 mg/mL and 62% of products at 20 mg/mL).

For nicotine pouches, there is considerable heterogeneity in the nicotine concentrations, ranging from 1.5 to 30 mg of nicotine per pouch.

## 4 Discussion

This study shows a profound and rapid transformation of the Swiss tobacco and nicotine products market from the mid-2000s. After a steady and moderate increase up until 2019, the number of notified products tripled to reach a record high in early 2026.

### 4.1 Cigars and cigarillos

One of the most notable findings is the extremely high number of cigars and cigarillos (more than 3,100 products), even though their prevalence of consumption remains low in Switzerland (around 2% among aged 15+) (13). A similar pattern has been observed in the EU, where cigars represented the largest category of notified tobacco products and were the most frequently notified tobacco product type in most countries analyzed (12). In Switzerland, this observation may be partly explained by 2 major regulatory exceptions for these products, including more favorable taxation (14) and the possibility of direct personal promotion (10). In addition, flavored products and descriptors that may be particularly appealing to young people were identified among several cigar and cigarillo products. Together, these findings suggest that cigar and cigarillo regulations deserve further attention within Swiss tobacco control policies.

### 4.2 Cigarettes

In contrast, the number of notified cigarettes is relatively low (237 products). The major tobacco companies control most of the supply, which limits the diversity of notified products. However, it is noteworthy that several supermarket groups are also positioned in this market. This is particularly the case with Denner, Coop and Lidl. While the number of cigarettes sold continues to decline over the years (2), this strategic positioning reflects the persistent strong demand for this product. In 2025, more than 15% of people aged 15+ smoked cigarettes, including 11% on a daily basis (15). Cigarettes also remain the most commonly consumed products overall (15, 16). This highlights that a limited number of product references does not necessarily reflect low market importance. In this context, the cigarette market remains undoubtedly lucrative, especially since taxation has not changed since 2013, while the average price per pack continues to rise (17).

### 4.3 Electronic cigarettes, nicotine pouches and snus

Electronic cigarettes raise significant concerns related to smoking cessation, health effects, youth appeal, as well as product regulation and advertising ban. These have been abundantly discussed elsewhere (1, 3, 7, 8, 18–23). The present study focused on the notified products in Switzerland.

Electronic cigarettes and refill liquids account for 51% of all notified products. As observed in other countries (24), flavor descriptors were dominated by fresh, fruity and menthol-related terms, whereas tobacco-related descriptors were less frequent. Several descriptors identified in product names (e.g., lemonade, cola, candy, bubble gum and mojito) may be particularly appealing to young people. Recent evidence indicates that non-tobacco flavors (e.g., fruit, sweet, cooling and mint/menthol) are commonly used by young people and may increase the appeal of electronic cigarettes and motivation to use these products (22, 23).

Nicotine concentrations in electronic cigarettes are relatively uniform (17–20 mg/mL), consistent with the maximum authorized nicotine content of 20 mg/mL (10). In contrast, as observed elsewhere (25), nicotine pouches vary greatly in nicotine content, ranging from 1.5 to 30 mg per pouch. Switzerland, like many other countries (26), only partially regulates nicotine pouches. Although these products are regulated by the Federal Act on Tobacco Products (e.g., prohibition of sale to minors, advertising restrictions, mandatory warnings) (2), this does not set a legal maximum limit. This regulatory asymmetry raises public health concerns: high and variable doses may promote faster nicotine addiction, particularly among young people exposed to flavored products. In Switzerland, nicotine pouches are also the cheapest form of nicotine delivery (21).

### 4.4 Limitations and perspectives

This study has two main limitations. Firstly, it deals with reported products and does not reflect sales, market share (27), consumption prevalence nor public health burden. In many countries, cigarettes remain the most widely sold product (28). Therefore, the number of notified products should not be interpreted as an indicator of consumption patterns or public health impact. Furthermore, it is plausible that the actual number of products on the Swiss market prior to the Federal Act on Tobacco Products was higher than official records reflect. As manufacturers and importers have 1 year to declare products sold, it is possible that some of them were released onto the market and then withdrawn if they sold poorly, did not comply with regulations (29) or were affected by regulatory changes (e.g., ban on the sale of nicotine products to minors, restrictions on the nicotine content of electronic cigarettes) (30, 31). As a result, the database may provide only a partial and time-bound representation of the market. In addition, a reported market launch date was available for only 76% of notified products. Consequently, historical trend analyses should be interpreted with caution, as products without a reported launch date could not be included in temporal analyses and may have influenced the reconstruction of long-term product diversification patterns. Another limitation concerns the completeness of notifications. The database relies on information submitted by manufacturers and importers and could not be independently verified against all products available on the Swiss market. For example, no heated tobacco products attributed to JTI were identified in the database extracted despite the company’s presence in this market segment. This discrepancy may reflect delayed, incomplete or incorrect data notification, or differences in company attribution. Additional verification would be required to determine the underlying reason. Consequently, the completeness of the database depends on industry compliance as well as regulatory oversight and enforcement activities.

Secondly, the available data does not provide information on where and how tobacco and nicotine products are marketed. In Switzerland, these products can be distributed through a wide range of retail channels, including supermarkets, kiosks, petrol stations, vending machines and online platforms. Their availability may also vary across different regions of the country (19). Beyond product names and flavors, the attractiveness of tobacco and nicotine products is also strongly shaped by marketing strategies, including advertising, promotion and sponsorship. However, there are currently no recent and systematic data documenting the marketing practices used to promote these products in Switzerland. This gap is particularly relevant considering ongoing regulatory changes.

The Federal Act on Tobacco Products, which entered into force in October 2024, introduced a nationwide ban on sales to minors and minimal advertising restrictions (2, 32). A revised version is expected to come into force in 2027 to introduce additional restrictions to ban tobacco advertising reaching children and youth (33). It will therefore no longer be possible to advertise in points of sale, newspapers, on the internet or on social media (unless access is restricted to adults). If implemented, this revised law is expected to reduce the visibility and promotion of tobacco and nicotine products. In this evolving regulatory context, some research initiatives are being developed to establish long-term monitoring of marketing strategies for tobacco and nicotine products in Switzerland (34). This will help to document how industry practices adapt to these new regulations and how such changes may shape market dynamics across products.

## 5 Conclusion

This study documents the rapid transformation of the Swiss tobacco and nicotine product landscape after a long period of relative stability. In terms of the number of notified products, the notified product landscape was historically dominated by cigars, despite their low prevalence of use. Since 2022, notifications of products have increased sharply, largely driven by the increase in notified electronic cigarette-related products. Although relatively few cigarette products are notified, they remain the first product consumed by the general population (15), representing a large and highly profitable market segment for tobacco companies. Electronic cigarettes, which represented more than half of all notified products, were frequently associated with flavor descriptors that may be particularly appealing to young people (6, 7, 20, 22, 23, 35).

The introduction of an electronic mandatory product notification system under the Federal Act on Tobacco Products since 2024 represents a major advance for market surveillance and regulatory oversight in Switzerland. However, missing products suggest compliance and enforcement gaps. The predominance of flavored products, the rapid diversification of electronic cigarettes and the wide variation in nicotine pouch concentrations highlight several areas that may require continued monitoring and regulatory evaluation. Strengthening the regulations – including removing exemptions for cigars and cigarillos, introducing maximum nicotine dose for nicotine pouches, increasing excise taxes, implementing plain packaging and ratifying the World Health Organization Framework Convention on Tobacco Control – would represent an important step towards a comprehensive, evidence-based tobacco control strategy to reduce tobacco and nicotine-related harms in Switzerland. By providing the first systematic overview of notified tobacco and nicotine products at the national level, this study offers a valuable evidence base to inform policy development, support prevention strategies and guide future monitoring efforts.

## Data Availability

Data from the electronic reporting and information system for tobacco products and electronic cigarettes are publicly available at Tabacinfo.ch.
