# Supplementary material for: Tobacco and nicotine products in Switzerland: a national analysis of market notifications
Source: Front Public Health. 2026 Oct 28;14:1876332. doi: 10.3389/fpubh.2026.1876332 (PMC13752828; doi:10.3389/fpubh.2026.1876332)
Supplement: Supplementary file 1 [file Table_1.docx]

Supplementary Material

**Supplementary Table 1.** List of the top 3 manufacturers and importers who notified the highest number of products by category (data from the official dataset Tabacinfo.ch, as of January 2026).

| **Product** | **Number of manufacturers and importers (N) and notified products (n)** | **1^st^** | **2^nd^** | **3^rd^** |
| --- | --- | --- | --- | --- |
| Cigarettes | N = 13  n = 237 | British American Tobacco Switzerland  (n = 101; 43 %) | Philip Morris Products SA  (n = 32; 14 %) | Japan Tobacco International AG Dagmersellen  (n = 28; 12 %) |
| Cigars | N = 20  n = 3,102 | The Royal Cigar Compagny AG (n = 547; 18 %) | Uniportis AG  (n = 473; 15 %) | Intertabak AG  (n = 466; 15 %) |
| Cigarillos | N = 9  n = 187 | Intertabak AG  (n = 67; 36 %) | Wellauer AG  (n = 32; 17 %) | Oettinger Davidoff AG  (n = 30; 16 %) |
| Herbal cigarettes | N = 2  n = 7 | Flowline AG  (n = 6; 86 %) | Elevated Epicurus Sàrl  (n = 1; 14 %) |  |
| Pipe tobacco | N = 10  n = 300 | Oettinger Davidoff AG  (n = 93; 31 %) | The Royal Cigar Compagny AG (n = 79; 26 %) | Urs Portmann Tabakwaren AG (n = 46; 15 %) |
| Roll-your-own tobacco | N = 13  n = 104 | Pölschl Tobacco Switzerland AG (n = 17; 16 %) | Wellauer AG  (n = 17; 16 %) | Japan Tobacco International AG Dagmersellen  (n = 16; 15 %) |
| Water pipe tobacco | N = 1  n = 19 | InSmoke AG  (n = 19; 100 %) |  |  |
| Heated tobacco products | N = 2  n = 46 | Philip Morris Products SA  (n = 25; 54 %) | British American Tobacco Switzerland  (n = 21; 46 %) |  |
| Herbal product for heating without tobacco | N = 5  n = 33 | First Class CBD (Schweiz) GmbH (n = 22; 67 %) | Green Passion GmbH  (n = 4; 12 %) | Oettinger Davidoff AG  (n = 4; 12 %) |
| Disposable cartridge/Pod with nicotine | N = 18  n = 688 | InSmoke AG  (n = 135; 20 %) | Vape GmbH  (n = 121; 18 %) | Playsure SA  (n = 91; 13 %) |
| Disposable cartridge/Pod without nicotine | N = 2  n = 37 | InSmoke AG  (n = 27; 73 %) | Playsure SA  (n = 10; 27 %) |  |
| Electronic cigarettes with nicotine | N = 20  n = 509 | InSmoke AG  (n = 155; 30 %) | Next Tröber AG (n = 60; 12 %) | Digital Heaven GmbH  (n = 50; 10 %) |
| Electronic cigarettes without nicotine | N = 9  n = 143 | Playsure SA  (n = 88; 62 %) | Evapify Switzerland SA (n = 17; 12 %) | Next Tröber AG (n = 16; 11 %) |
| Refill liquid with nicotine | N = 20  n = 2,065 | Playsure SA  (n = 889; 43 %) | InSmoke AG  (n = 408; 20 %) | Viviswiss GmbH (n = 280; 14 %) |
| Refill liquid without nicotine | N = 22  n = 1,590 | Playsure SA  (n = 668; 42 %) | Vapo-Premium Sàrl  (n = 181; 11 %) | Vendex AG  (n = 168; 11 %) |
| Chewing tobacco | N = 3  n = 6 | Oettinger Davidoff AG  (n = 4; 67 %) | Burger Söhne SA (n = 1; 17 %) | Next Tröber AG (n = 1; 17 %) |
| Nicotine pouches | N = 11  n = 162 | British American Tobacco Switzerland  (n = 28; 17 %) | Trompi GmbH  (n = 26; 16 %) | Next Tröber AG (n = 23; 14 %) |
| Snus | N = 2  n = 32 | Next Tröber AG (n = 28; 88 %) | Eleven Partners SA  (n = 4; 13 %) |  |
| Cannabis with low THC content | N = 44  n = 549 | Swiss Botanic GmbH  (n = 59; 11 %) | Zauber - Blüten Hanf GmbH  (n = 52; 9 %) | Genuine Swiss GmbH  (n = 42; 8 %) |
| Snuff with tobacco | N = 5  n = 41 | Pölschl Tobacco Switzerland AG (n = 17; 41 %) | Oettinger Davidoff AG  (n = 12; 29 %) | Wellauer  (n = 7; 17 %) |
